# Role of integrin-linked kinase in regulating the protein stability of the MUC1-C oncoprotein in pancreatic cancer cells

**DOI:** 10.1038/oncsis.2017.61

**Published:** 2017-07-10

**Authors:** H-L Huang, H-Y Wu, P-C Chu, I-L Lai, P-H Huang, S K Kulp, S-L Pan, C-M Teng, C-S Chen

**Affiliations:** 1 The PhD Program for Cancer Biology and Drug Discovery, College of Medical Science and Technology, Taipei Medical University, Taipei, Taiwan; 2 Division of Medicinal Chemistry and Pharmacognosy, College of Pharmacy, The Ohio State University, Columbus, OH, USA; 3 Institute of Biological Chemistry, Academia Sinica, Taipei, Taiwan; 4 Institute of Biochemical Science, National Taiwan University, Taipei, Taiwan; 5 Epigenome Research Center, China Medical University Hospital, Taichung, Taiwan; 6 Department of Biochemistry and Molecular Biology, College of Medicine, National Cheng Kung University, Tainan, Taiwan; 7 Department of Pharmacology, College of Medicine, Taipei Medical University, Taipei, Taiwan; 8 Pharmacological Institute, College of Medicine, National Taiwan University, Taipei, Taiwan

## Abstract

MUC1-C overexpression has been associated with the progression of pancreatic tumors by promoting the aggressive and metastatic phenotypes. As *MUC1* is a STAT3 target gene, STAT3 plays a major role in regulating MUC1-C expression. In this study, we report an alternative mechanism by which integrin-linked kinase (ILK) post-transcriptionally modulates the expression of MUC1-C by maintaining its protein stability in pancreatic cancer cells. We found that ILK acts in concert with STAT3 to facilitate IL-6-mediated upregulation of MUC1-C; ILK depletion was equally effective as STAT3 depletion in abolishing IL-6-induced MUC1-C overexpression without disturbing the phosphorylation or cellular distribution of STAT3. Conversely, ectopic expression of constitutively active ILK increased MUC1-C expression, though this increase was not noted with kinase-dead ILK. This finding suggests the requirement of the kinase activity of ILK in regulating MUC1-C stability, which was confirmed by using the ILK kinase inhibitor T315. Furthermore, our data suggest the involvement of protein kinase C (PKC)δ in mediating the suppressive effect of ILK inhibition on MUC1-C repression. For example, co-immunoprecipitation analysis indicated that ILK depletion-mediated MUC1-C phosphorylation was accompanied by increased phosphorylation of PKCδ at the activation loop Thr-507 and increased binding of PKCδ to MUC1-C. Conversely, ILK overexpression resulted in decreased PKCδ phosphorylation. From a mechanistic perspective, the present finding, together with our recent report that ILK controls the expression of oncogenic KRAS through a regulatory loop, underscores the pivotal role of ILK in promoting pancreatic cancer progression.

## Introduction

Mucin 1 (MUC1), a heterodimeric transmembrane glycoprotein, is one of the mucin family members that form protective mucous barriers on epithelial surfaces.^1^ The *MUC1* gene encodes a single polypeptide chain that is auto-proteolytically cleaved to generate two subunits, the N-terminal subunit (MUC1-N) and the C-terminal subunit (MUC1-C), which form a stable complex.^2, 3^ Dysregulated expression of MUC1 has been associated with the progression of many types of epithelial tumors due to its myriad oncogenic functions. In addition to its protective and lubricative functions for the underlying epithelia, MUC1 also regulates diverse cellular functions that promote the aggressive and metastatic phenotypes of cancer cells through an intricate interplay of the MUC1-C subunit with various signaling effectors at different subcellular locales.^3, 4, 5, 6, 7^ At the cell membrane, MUC1-C interacts with epidermal growth factor receptor and other receptor tyrosine kinases, leading to the activation of downstream signaling cascades.^3, 6, 8^ Moreover, cytoplasmic MUC1-C can be translocalized into the nucleus and mitochondria via distinct mechanisms, where it activates multiple nuclear signaling pathways and blocks activation of the intrinsic apoptotic pathway, respectively.^3, 5, 6^ Particularly noteworthy is the role of MUC1-C as a regulator of cell metabolism, which confers resistance to hypoxia and metabolic stress upon cancer cells.^9^ Consequently, MUC1 overexpression in the mammary gland results in breast tumorigenesis in the MMTV–MUC1 transgenic model.^10^ Taken together, MUC1-C represents a clinically relevant target for cancer therapy,^5, 6, 8^ with the proof of concept provided by the *in vivo* efficacy of different peptide inhibitors of MUC1-C heterodimerization, including PMIP^11^ and GO-201/203,^12, 13, 14^ in suppressing xenograft tumor growth in different animal models. Moreover, GO-203-2c is currently undergoing phase I evaluation in patients with acute myeloid leukemia (https://clinicaltrials.gov/ct2/show/NCT02204085).

There is evidence that cancer cells adopt multiple strategies, at the transcriptional, post-transcriptional and epigenetic levels, to regulate MUC1-C expression to gain growth advantage.^5, 15, 16, 17, 18, 19^ The mucin gene *MUC1* was reported to be a target gene of STAT3 in various cancer cell lines.^20, 21^ Especially noteworthy is a reported autoinductive loop between MUC1-C and STAT3 in breast cancer cells, in which MUC1-C facilitates JAK1-mediated STAT3 activation through complex formation, leading to activation of *MUC1* gene.^22^ Furthermore, evidence suggests that peroxisome proliferator-activated receptor (PPAR)γ has a dichotomous role in regulating MUC1 expression in a cell type/context-specific manner. For example, while liganded PPARγ stimulates *MUC1* induction in murine trophoblasts,^23^ it was reported to antagonize progesterone-mediated MUC1 expression in human uterine epithelial cell lines and T47D breast cancer cell lines.^24^ Moreover, PPARγ acts as an E3 ligase to facilitate MUC1-C degradation in HT-29 and MCF-7 cells independent of its transcriptional activity.^25^ In addition to gene amplification and transcriptional activation,^6^ breast cancer cells might also increase MUC1-C expression by abrogating the expression of miR-125b or miR-145, which suppressed the translation of MUC1 by binding to the *MUC1* 3′UTR.^17, 26^

Integrin-linked kinase (ILK) is a serine/threonine kinase that exhibits a multitude of oncogenic functions in promoting carcinogenesis and tumor progression in different cancer types.^27, 28, 29^ For example, in pancreatic cancer, ILK regulates tumor growth via the activation of the Akt and STAT3 signaling pathways,^30^ and plays a key role in IL-1β-induced enhancement of adhesion and invasion through a p38 MAP kinase-dependent pathway.^31^ More recently, our laboratory demonstrated a KRAS–ILK regulatory loop that links ILK to the regulation of oncogenic KRAS expression.^32^ In this study, we report an alternative mechanism for ILK to post-transcriptionally regulate the expression of MUC1-C by maintaining MUC1-C protein stability in pancreatic cancer cells, which, in conjunction with STAT3, is responsible for IL-6-mediated upregulation of MUC1-C. Specifically, ILK negatively regulated protein kinase C (PKC)δ, which facilitated the phosphorylation and subsequent proteasomal degradation of MUC1-C. In consequence, MUC1-C expression could be suppressed by genetic depletion or pharmacological inhibition of ILK through protein destabilization. From a mechanistic perspective, the unique ability of ILK to regulate MUC1-C stability, in conjunction with STAT3-activated *MUC1* gene expression,^22^ upregulates MUC1-C and enables pancreatic cancer cells to interact with the tumor microenvironment to promote an aggressive phenotype.

## Results

### ILK mediated the effect of IL-6 on MUC1-C overexpression concurrently with STAT3 activation

**Figure 1:**
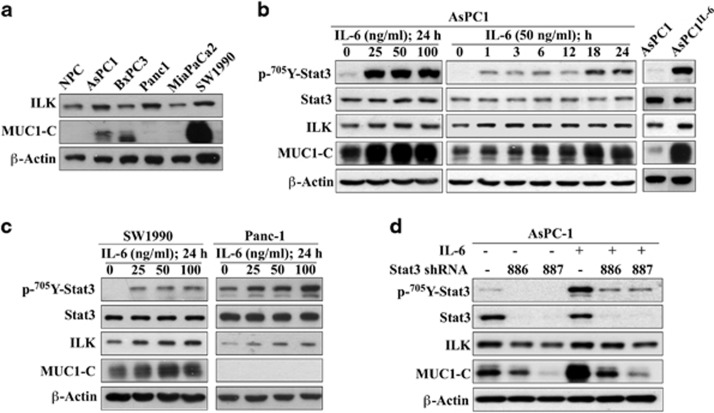
IL-6 stimulates MUC1-C expression in pancreatic cancer cells, in part through STAT3 activation. (**a**) Western blot analysis of the basal expression levels of ILK and MUC1-C in NPCs versus five pancreatic cancer cell lines. (**b**) Concentration- (left) and time-dependent (center) effect of exogenous IL-6 and stable expression of IL-6 (right) on the phosphorylation and/or expression levels of STAT3, ILK and MUC1-C in AsPC-1 cells. (**c**) Concentration-dependent effect of IL-6 on the phosphorylation and/or expression levels of STAT3, ILK and MUC1-C in MUC1-overexpressing SW1990 and MUC1-deficient Panc-1 cells. (**d**) Effect of STAT3 knockdown by two different shRNAs (#886 and #887) on the phosphorylation and/or expression of STAT3, ILK and MUC1-C in AsPC-1 cells without or with the treatment with 50 ng/ml IL-6 for 24 h. Data shown are representative of at least three independent experiments.

**Figure 2:**
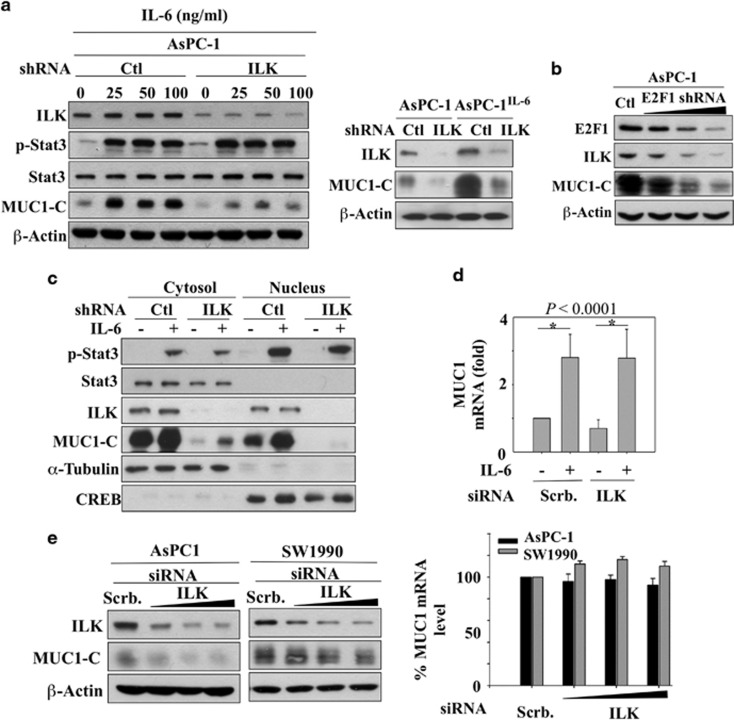
sh/siRNA-mediated knockdown of ILK suppresses endogenous and IL-6-stimulated upregulation of MUC1-C expression without altering MUC1 mRNA expression. (**a**) Effect of shRNA-mediated silencing of ILK versus control on the phosphorylation and/or expression of STAT3 and MUC1-C in IL-6-treated AsPC-1 or AsPC-1^IL-6^ cells. For ILK depletion, AsPC-1 cells were transfected with ILK shRNA using lentivirus infection to generate a stable knockdown clone. For exogenously added IL-6, cells were treated with IL-6 at the indicated concentrations for 24 h. (**b**) shRNA-mediated knockdown of E2F1 suppressed the expression of ILK and MUC1-C in AsPC-1 cells. (**c**) The suppressive effect of ILK depletion on endogenous and IL-6-induced upregulation of MUC1-C expression is independent of STAT3. ILK silencing did not affect the cellular distribution and phosphorylation status of STAT3 in AsPC-1 cells. The stable ILK-depleted cells were treated with vehicle or IL-6 (50 ng/ml) for 24 h. (**d**) qRT-PCR analysis of the effect of siRNA-mediated ILK depletion versus control on MUC1 mRNA expression in IL-6- versus vehicle-treated AsPC-1 cells (means±s.e., *n*=3). Scrb, scrambled (control) siRNA. Cells were transiently transfected with ILK siRNA for 48 h followed by treatment with IL-6 (50 ng/ml) for an additional 24 h, and subjected to qRT-PCR analysis. (**e**) Left, effect of siRNA-mediated knockdown of ILK on the expression of endogenous MUC1-C in AsPC-1 and SW1990 cells. Cells were transfected with ILK siRNA for 72 h. Right, qRT-PCR analysis of the effect of ILK depletion versus control on MUC1 mRNA expression in AsPC-1 and SW1990 cells (means±s.e., *n*=5 and 3, respectively). Cells underwent the same treatment before qRT-PCR analysis for MUC1 mRNA expression. In western blot analysis, data shown are representative of at least three independent experiments.

We measured MUC1-C expression in normal pancreatic cells (NPCs) versus different pancreatic cancer cell lines by western blot analysis. While NPCs, BxPC-3, Panc-1 and MiaPaCa2 cells lacked appreciable MUC1-C expression, AsPC-1 and SW1990 cells exhibited low and high abundance of MUC1-C, respectively (Figure 1a). Consistent with the reported effect of IL-6 on MUC1 overexpression in breast and lung cancer cells,^22, 33^ exposure of AsPC-1 cells to IL-6 led to parallel increases in STAT3 phosphorylation and the expression of MUC1-C, and, to a lesser extent, ILK (Figure 1b). The ability of IL-6 to stimulate MUC1-C expression was confirmed by the stable expression of IL-6 in AsPC-1 (AsPC-1^IL-6^) cells (Figure 1b, right panel) and in IL-6-treated SW1990 cells (Figure 1c, left). This phenomenon, however, was not noted in MUC1-deficient Panc-1 cells (Figure 1c, right),

As *MUC1* is a STAT3 target gene,^20, 21, 22, 33^ it follows that STAT3 activation played a crucial role in regulating MUC1-C expression in AsPC-1 cells. STAT3 depletion by two different shRNAs (#886 and #887) was effective in reducing the expression of endogenous MUC1-C as well as in blocking IL-6-induced MUC1-C upregulation (Figure 1d). In parallel, we also examined the effect of shRNA-mediated ILK knockdown on MUC1 expression in AsPC-1 cells treated with exogenous IL-6 or stably expressing IL-6. It is noteworthy that ILK depletion was equally as effective as STAT3 depletion in abolishing IL-6-induced MUC1-C overexpression (Figure 2a). Similarly, shRNA-mediated knockdown of E2F1, a transcription factor that regulates ILK gene expression,^32, 34^ led to parallel decreases in MUC1-C levels in AsPC-1 cells (Figure 2b). In order to rule out the possibility that the inhibitory effect of ILK knockdown on MUC1-C expression might be associated with its ability to interfere with STAT3 signaling, we fractionated cellular and nuclear fractions to determine ILK knockdown affect the phosphorylation and/or cellular distribution of STAT3 in response to IL-6. However, this ILK depletion did not cause appreciable changes in the phosphorylation or cellular distribution of STAT3 in IL-6-treated AsPC-1 cells (Figure 2c), indicating that this suppressive effect was attributable to the cooperative interplay between ILK and STAT3. Moreover, although silencing of ILK caused a sharp decrease in MUC1-C abundance, the mRNA expression of MUC1 remained unaltered (Figure 2d). Furthermore, ILK knockdown in untreated AsPC-1 and SW1990 cells caused downregulation of endogenous MUC1-C expression without affecting MUC1 mRNA levels (Figure 2e). We also found ILK knockdown to have the same effect on MUC1-C expression in two other cancer cell lines, including DU-145 prostate cancer and MDA-MB-231 breast cancer cells (Supplementary Figure S1), revealing that the suppressive effect of ILK depletion on MUC1-C expression was not a cell line-specific phenomenon. Together, these findings demonstrated the unique ability of ILK to regulate MUC1-C expression independently of MUC1 gene expression.

**Figure 3:**
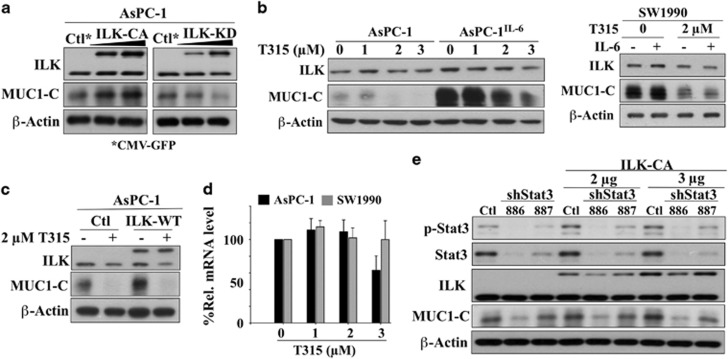
Evidence that ILK kinase activity is involved in regulating MUC1-C expression. (**a**) Ectopic expression of GFP-tagged constitutively active ILK (ILK-CA), but not GFP-tagged kinase-dead ILK (ILK-KD), mimics the effect of IL-6 on MUC1-C upregulation in AsPC-1 cells. (**b**) Suppressive effect of T315 on (left) MUC1-C expression in AsPC-1 and AsPC-1^IL-6^ cells, and (right) endogenous and IL-6-induced upregulation of MUC1-C expression in SW1990 cells. SW1990 cells were exposed to IL-6 (50 ng/ml) and T315 (2 μM) concurrently for 24 h. (**c**) T315 (2 μM) was effective in suppressing wild-type ILK (ILK-WT)-induced overexpression of MUC1-C in AsPC-1 cells. (**d**) qRT-PCR analysis of the dose-dependent effect of T315 versus control on MUC1 mRNA expression in AsPC-1 and SW1990 cells (means±s.e., *n*=3). (**e**) Lack of effect of the ectopic expression of GFP-tagged ILK-CA on STAT3 knockdown-induced depletion of MUC1-C expression in AsPC-1 cells. In western blot analysis, data shown are representative of at least three independent experiment.

The mechanistic link between ILK and MUC1-C expression was further confirmed by the ability of ectopically expressed constitutively active ILK to increase MUC1-C expression in AsPC-1 cells (Figure 3a, left). This upregulation, however, did not occur when the kinase-dead form of ILK was overexpressed (right), suggesting that the kinase activity of ILK was required to mediate the effect on MUC1-C expression. Pursuant to this finding, we used an ILK kinase inhibitor, T315,^32, 35^ as a proof of concept to verify the functional role of ILK. Consistent with the consequences of ILK depletion, exposure of AsPC-1, AsPC-1^IL-6^ and SW1990 cells to T315 suppressed the expression of endogenous MUC1-C and inhibited IL-6-induced MUC1-C overexpression (Figure 3b). Furthermore, T315 (2 μM) was effective in abrogating the effect of ILK overexpression on MUC1-C expression (Figure 3c). Quantitative real-time PCR (qRT-PCR) analysis indicated that MUC1 mRNA expression levels remained largely unaltered by T315 in AsPC-1 and SW1990 cells, with the exception of T315 at 3 μM in AsPC-1 cells (Figure 3d), which might be attributed to extensive cell death. Nevertheless, overexpression of ILK-CA failed to increase MUC1-C levels in STAT3-depleted AsPC-1 cells, suggesting that ILK acted downstream of STAT3 in regulating MUC1-C expression (Figure 3e).

In addition to IL-6, hypoxia also induces MUC1-C accumulation in pancreatic cancer cells, in turn driving hypoxia-driven angiogenesis.^36^ Consistent with this report, subjecting AsPC-1 cells to hypoxic conditions led to MUC1-C upregulation in a time-dependent manner, which could be reversed to the basal level by T315 treatment (Supplementary Figure S2).

### ILK inhibition promotes ubiquitin-dependent MUC1-C degradation

**Figure 4:**
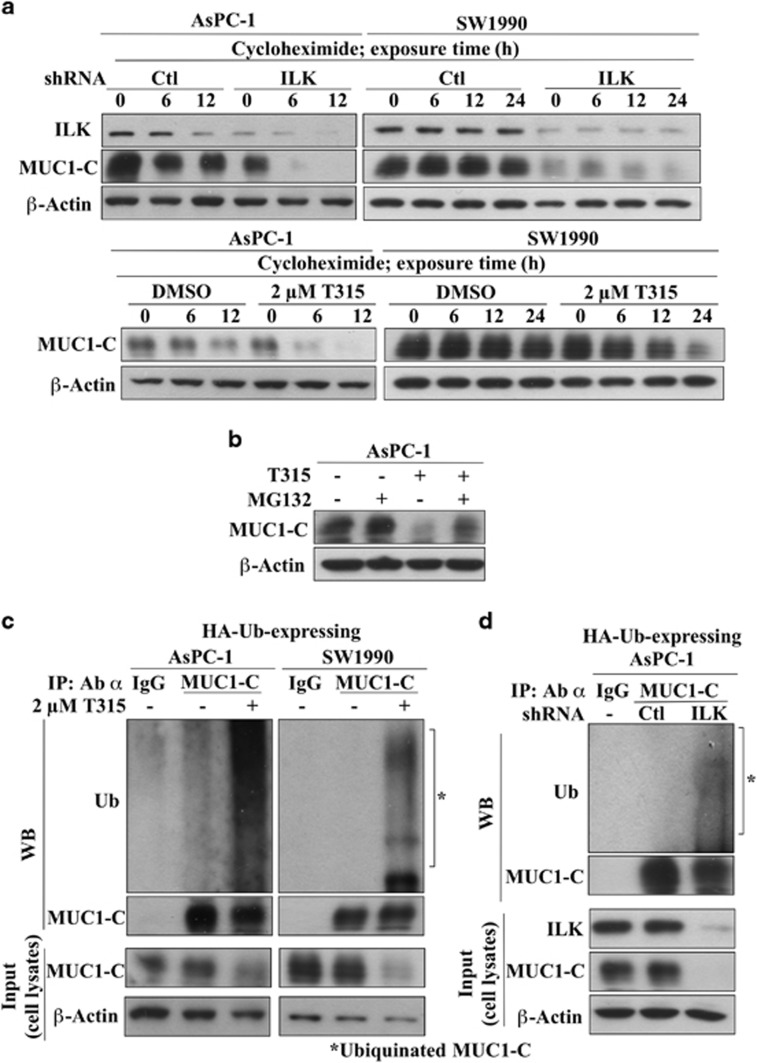
ILK inhibition facilitated the suppression of MUC1-C expression through ubiquitin-dependent proteasomal degradation. (**a**) T315 (upper) or shRNA-mediated ILK knockdown (lower) decreased the protein stability of MUC1-C in AsPC-1 and SW1900 cells. Cells were co-treated with T315 (2 μM) and cycloheximide (100 μg/ml) or ILK-depleted cells were treated with cycloheximide (100 μg/ml) for the indicated time intervals. (**b**) The proteasome inhibitor MG-132 protects AsPC-1 and SW1990 cells from T315-mediated MUC1-C degradation. Cells were treated with 1 μM MG-132 and 2 μM T315, individually or in combination, versus vehicle control for 24 h. (**c**, **d**) ILK inhibition via T315 treatment or shRNA-mediated ILK depletion facilitates MUC1-C ubiquitination in AsPC-1 and/or SW1990 cells. Cells ectopically expressing HA-ubiquitin (HA-Ub) were treated with (**c**) 2 μM T315 for 18 h followed by co-treatment with 1 μM MG-132 for an additional 6 h or (**d**) 1 μM MG-132 for 6 h. Equal amounts of cell protein lysates were immunoprecipitated with MUC1-C antibody and protein A/G agarose beads followed by immunoblotting with Ub and MUC1-C antibodies. Data shown are representative of at least three independent experiments.

On the basis of the qRT-PCR evidence that genetic knockdown or pharmacological inhibition of ILK did not alter MUC1 mRNA levels, we hypothesized that the effect of ILK inhibition on MUC1-C downregulation was attributable to proteasomal degradation, which was supported by the following studies. First, we used pulse chase assays to analyze the effect of ILK inhibition on MUC1-C protein stability. In the presence of cycloheximide, shRNA-mediated depletion of ILK (Figure 4a, upper) or T315 (lower) promoted the clearance of MUC1-C, relative to the DMSO control, in both AsPC-1 and SW1990 cells. Second, the proteasome inhibitor MG-132 protected AsPC-1 cells against the suppressive effect of T315 on MUC1-C expression (Figure 4b). Third, we used co-immunoprecipitation (Co-IP) analysis to investigate the effect of ILK inhibition on MUC1-C ubiquitination. In AsPC-1 and SW1990 cells ectopically expressing HA-tagged ubiquitin (HA-Ub), the ability of T315 (2 μM) or genetic ILK knockdown to suppress MUC1-C expression was attributable to increased MUC1-C ubiquitination (Figures 4c and d). Together, these data reveal the pivotal role of ILK in the regulation of MUC1-C protein stability.

### PKCδ is involved in mediating the ubiquitin-dependent degradation of MUC1-C in response to ILK inhibition

**Figure 5:**
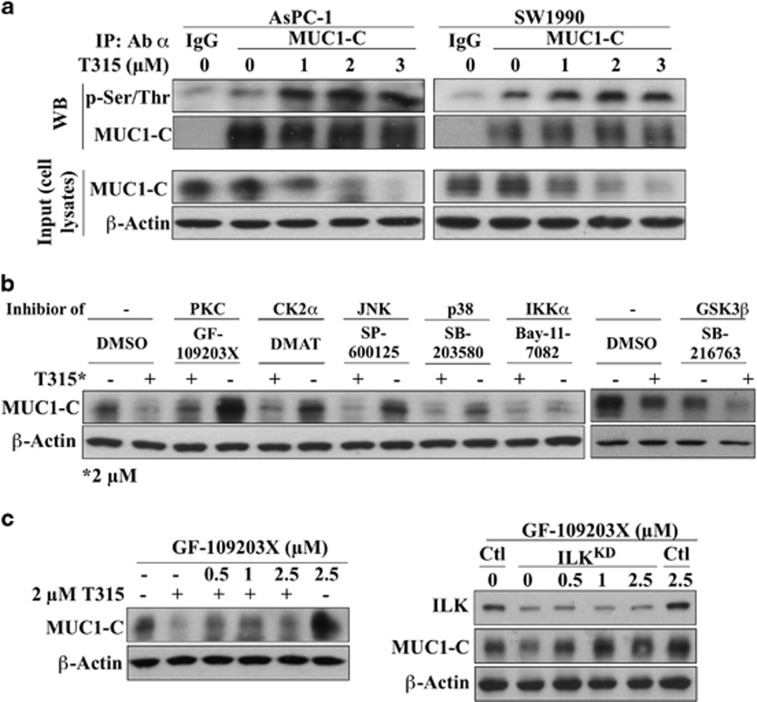
A pharmacological approach to identify the kinase responsible for ILK inhibition-mediated MUC1-C degradation. (**a**) Co-IP analysis of the effect of T315 on MUC1-C phosphorylation in AsPC-1 and SW1990 cells. Cells were treated with T315 at the indicated concentrations for 24 h. Equal amounts of cell lysates were immunoprecipitated with MUC1-C antibody and protein A/G agarose beads followed by immunoblotting with p-Ser/Thr and MUC1-C antibodies. (**b**) Effects of different kinase inhibitors, including those for PKCs (GF-109203X, 5 μM), CK2α (DMAT, 2 μM), JNK (SP-600125, 5 μM), p38 (SB-203580, 2 μM), IKKα (BAY-11-7082, 2 μM) and GSK3β (SB-216763, 10 μM), on T315-induced MUC1-C downregulation in AsPC-1 cells after co-treatment for 24 h. (**c**) Dose-dependent protective effect of GF-109293X against MUC1-C degradation induced by 2 μM T315 (left) or shRNA-mediated ILK knockdown (right) in AsPC-1 cells after co-treatment of 2 μM T315 and indicated concentration of GF-109203X for 24 h. Data shown are representative of at least three independent experiments.

As ubiquitin-dependent protein degradation is preceded by phosphorylation,^37^ we evaluated the effect of T315 on MUC1-C phosphorylation via Co-IP. As shown, concentration-dependent downregulation of MUC1-C expression was associated with parallel increases in Ser/Thr phosphorylation in both AsPC-1 and SW1990 cells (Figure 5a). Although MUC1-C is known to be phosphorylated by multiple kinases,^8^ including glycogen synthase kinase (GSK)3β and PKCδ,^38, 39^ the identity of the kinase responsible for its degradation remained undefined. Thus, we analyzed a panel of kinase inhibitors for the ability to block T315-induced suppression of MUC1-C expression in AsPC-1 cells, including the pan-PKC inhibitor GF-109203X, the casein kinase (CK)2α inhibitor DMAT, the c-Jun N-terminal kinase (JNK) inhibitor SP-600125, the p38 MAP kinase inhibitor SB-203580, the IκB kinase (IKK)α inhibitor Bay-11-7082 and the GSK3β inhibitor SB-216763. It is noteworthy that GF-109203X, when used alone, increased MUC1-C accumulation, and was able to partially protect cells from the suppressive effect of T315, and, to a greater extent, ILK knockdown on MUC1-C expression (Figures 5b and c). In contrast, none of the other kinase inhibitors examined exhibited the ability to rescue cells from T315-mediated MUC1-C downregulation (Figure 5b).

**Figure 6:**
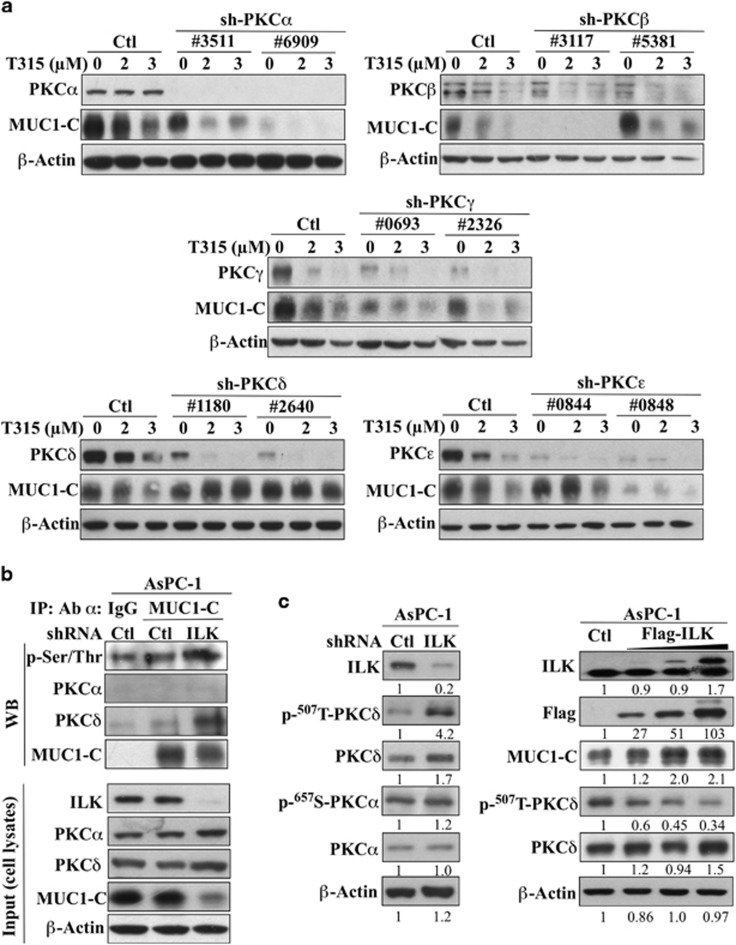
PKCδ is involved in facilitating T315 and ILK knockdown-mediated MUC1 phosphorylation and degradation. (**a**) Effects of shRNA-mediated selective depletion of PKC isoforms α, β, γ, δ and ε (two different shRNAs each) on T315-mediated MUC1-C downregulation. Selective PKC isoform-depleted cells were treated with T315 at the indicated concentrations for 24 h. (**b**) Co-IP analysis of the effect of shRNA-mediated ILK knockdown (left) and T315 (2 μM) treatment for 24 h (right) on MUC1-C phosphorylation and binding with PKCδ in AsPC-1 cells. PKCα was used as a negative control. Equal amounts of cell protein lysates were immunoprecipitated with MUC1-C antibody and protein A/G agarose beads followed by immunoblotting with p-Ser/Thr, PKCα, PKCδ and MUC1-C antibodies. (**c**) ILK negatively regulates PKCδ activation. Effects of shRNA-mediated knockdown (left) and ectopic expression of ILK (right) on PKCδ and/or PKCα phosphorylation. AsPC-1 cells were transiently transfected with Flag-WT-ILK for 48 h (right). Quantification was done by using ImageJ. Double bands shown in ILK and Flag were quantified separately and summed up together to get fold change compared to the control group. Data shown are representative of at least three independent experiments.

To identify the PKC isozyme responsible for MUC1-C degradation, we selectively depleted individual isozymes (α, β, γ, δ and ε) with two different shRNAs each and assessed the effects on T315-facilitated suppression of MUC1-C expression in AsPC-1 cells. Among these five isozymes, only knockdown of PKCδ resulted in the desired protective effect on MUC1-C repression, while silencing of the other isozymes had no significant appreciable protection (Figure 6a). The intermediary role of PKCδ in mediating this cellular response was further confirmed by the following experiments in AsPC-1 cells. First, Co-IP analysis revealed that ILK knockdown-mediated phosphorylation of MUC1-C was accompanied by a parallel increase in the binding of PKCδ to MUC1-C. This increased binding, however, was not noted with the negative control PKCα (Figure 6b). Second, shRNA-mediated depletion of ILK led to increased phosphorylation of PKCδ at the activation loop Thr-507, while PKCα phosphorylation remained unaltered (Figure 6c, left). Conversely, overexpression of ILK in AsPC-1 cells resulted in the reversal of the above changes in MUC1-C expression and Thr-507-PKCδ phosphorylation (Figure 6c, right). Taken together, these data suggest that PKCδ is regulated by ILK inhibition, as well as involved in ILK-mediated ubiquitin-dependent MUC1-C degradation.

**Figure 7:**
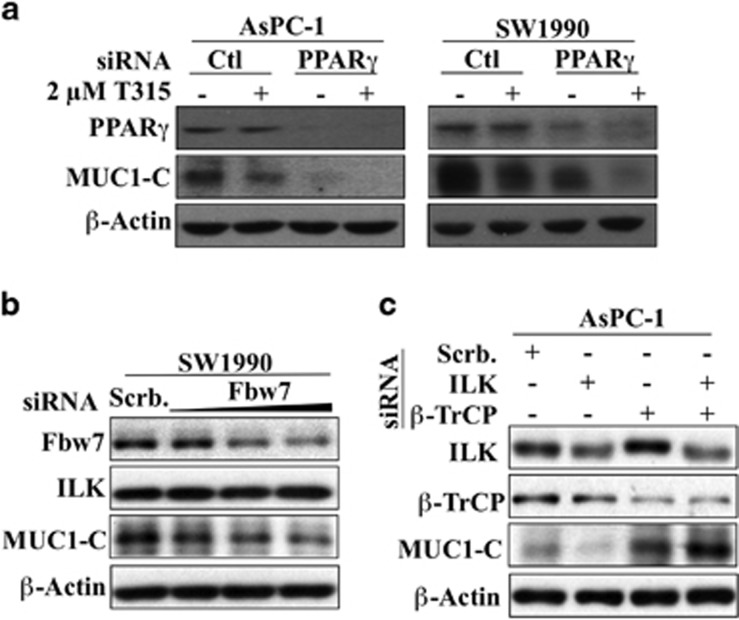
β-TrCP, not PPARγ, might be involved in ILK inhibition-induced MUC1-C degradation. (**a**) PPARγ depletion negatively impacted endogenous MUC1-C expression and could not protect T315-mediated downregulation of MUC1-C in AsPC-1 and SW1990 cells after 24 h of treatment. (**b**) Dose-dependent knockdown of Fbw7 induced a parallel reduction in MUC1-C expression in SW1990 cells, while ILK expression was unaffected. (**c**) Effect of β-TrCP knockdown on endogenous MUC1-C expression and ILK silencing-induced MUC1-C degradation in AsPC-1 cells. Data shown are representative of at least three independent experiments.

Pursuant to a recent report that PPARγ acts as an E3 ligase responsible for MUC1-C degradation independent of its transcriptional activity in HT-29 and MCF-7 cells,^25^ we investigated the potential involvement of PPARγ in T315-induced MUC1-C repression by examining whether siRNA-mediated knockdown of PPARγ could protect cells from this drug-induced MUC1-C degradation. However, silencing of PPARγ alone caused suppression of MUC1-C expression, and, together with T315, completely depleted MUC1-C in AsPC-1 and SW1990 cells (Figure 7a). Together, these data argued against the involvement of PPARγ as an E3 ligase in facilitating T315-mediated MUC1-C degradation in these pancreatic cancer cells.

To identify the E3 ligase responsible for ILK inhibition-induced MUC1-C degradation, we turned our attention to Fbw7 (F-box and WD repeat domain-containing 7; aka, Fbxw7) and β-TrCP in light of their tumor-suppressive activities in facilitating the degradation of oncogenic proteins.^40^ Accordingly, we analyzed the effects on MUC1-C expression when we depleted Fbw7 in SW1990 cells and β-TrCP in AsPC-1 cells, considering the endogenous levels of these E3 ligases in these two cell lines. In principle, silencing of the responsible E3 ligase should lead to the accumulation of MUC1-C. As shown, a dose-dependent reduction in Fbw7 expression caused a parallel decrease in MUC1-C levels (Figure 7b), while silencing of β-TrCP not only substantially increased the expression of MUC1-C, but also protected AsPC-1 cells from ILK depletion-induced downregulation of MUC1-C expression. Together, these data suggest the potential involvement of β-TrCP in mediating the ubiquitin-dependent degradation of MUC1-C, which warrants further investigation.

### *In vivo* mechanistic validation

**Figure 8:**
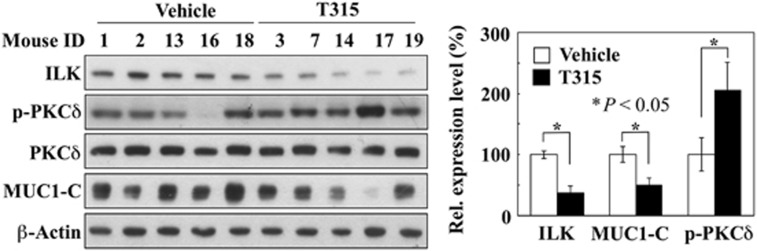
Western blot analysis of the effect of daily oral administration of T315 at 50 mg/kg on the expression of ILK, p-PKCδ, PKCδ and MUC1-C in AsPC-1 xenograft tumors in nude mice. Drug- versus vehicle-treated tumors were obtained in our previous study on the role of ILK in regulating KRAS expression,^32^ in which T315 treatment led to 51% suppression of tumor growth (described in Supplementary Figure S2; http://www.nature.com/onc/journal/v35/n30/extref/onc2015458x1.pdf).

To confirm our findings of the functional role of ILK in mediating PKCδ-dependent MUC1-C degradation *in vivo*, we examined the expression levels of relevant biomarkers in T315-treated AsPC-1 tumors after 21 days of treatment. These tumor samples were previously prepared in our recent study of the KRAS–ILK signaling loop, in which daily oral administration of T315 at 50 mg/kg inhibited AsPC-1 xenograft tumor growth by 51% (described in the Supplementary Figure S2 of cited reference;^32^ http://www.nature.com/onc/journal/v35/n30/extref/onc2015458x1.pdf). Consistent with the *in vitro* finding, T315 significantly downregulated ILK and MUC1-C expression and increased PKCδ phosphorylation in AsPC-1 tumors in nude mice (*P*<0.05; Figure 8).

## Discussion

Substantial evidence indicates that MUC1-C signaling is upregulated in the course of tumor progression to promote an aggressive phenotype, in part, through crosstalk with the tumor microenvironment.^5, 6, 8^ For example, a number of stress signals, including IL-6 and hypoxia, have been reported to induce the accumulation of MUC1-C to drive epithelial-mesenchymal transition (EMT) and angiogenesis.^22, 36^ Multiple mechanisms at different molecular levels have been reported to regulate MUC1-C overexpression,^5, 15, 16, 17, 18, 19^ among which the MUC1-C–STAT3 autoinductive loop in breast cancer cells is especially noteworthy.^22^ Here we report a post-transcriptional mechanism, in which ILK acts by blocking PKCδ-mediated protein degradation of MUC1-C. From a mechanistic perspective, the cooperative interplay between STAT3 and ILK would facilitate an immediate cellular response to IL-6 and other stress signals to upregulate MUC1-C expression at both transcriptional and post-transcriptional levels. As overexpression of ILK could not rescue the effect of STAT3 depletion on MUC1-C expression, it is likely that ILK acts downstream of STAT3 in MUC1-C regulation. Nevertheless, genetic knockdown or pharmacological inhibition of ILK caused depleted MUC1-C expression in IL-6-treated pancreatic cancer cells, indicating that ILK is required to sustain STAT3-induced MUC1 upregulation by maintaining its stability.

Moreover, in light of the ability of ILK to regulate oncogenic KRAS signaling,^32^ the present study also provides a mechanistic link between KRAS and MUC1-C. As MUC1-C has been reported to confer KRAS independence in mutant KRAS lung cancer cells,^41^ it warrants investigation whether targeting ILK can mitigate the effect of MUC1-C overexpression on KRAS independence.

Evidence suggests that T315-mediated MUC1-C degradation was facilitated by PKCδ-mediated phosphorylation (Figure 6). This finding is consistent with the report that PKCδ binds and phosphorylates MUC1-C on Thr-41 at the TDRSPY motif, which attenuates the interaction between MUC1-C and β-catenin.^39^ As PKCδ is activated in response to oxidative or genotoxic stress to trigger apoptotic responses,^42, 43, 44^ we hypothesize that activation of PKCδ by genetic depletion of ILK or T315 was attributable to reactive oxygen species (ROS) production and/or DNA damage, which is currently under investigation.

In contrast to the reported function of PPARγ as the E3 ligase responsible for MUC1-C degradation independent of its transcriptional activity in HT-29 and MCF-7 cells,^25^ our data refute this mechanistic link, as PPARγ depletion reduced endogenous MUC1-C expression and could not protect cells from T315-induced depletion of MUC1-C. This discrepancy might be attributable to differences in cell lines and cellular contexts between these two studies. In the present study, we obtained evidence that β-TrCP might be involved in ILK depletion-induced MUC1-C degradation. This has prompted further confirmation and is under investigation.

In summary, because ILK is activated in response to IL-6 and other environmental signals,^34, 45^ the unique ability of ILK to regulate MUC1-C stability, in conjunction with STAT3-activated MUC1 gene expression,^22^ enables pancreatic cancer cells to interact with stress signals in the tumor microenvironment, such as IL-6 and hypoxia. The interactions promote an aggressive phenotype, in part through MUC1-C upregulation. From a mechanistic perspective, the present finding, together with our recent report that ILK controls the expression of oncogenic KRAS through a regulatory loop,^32^ underscores the pivotal role of ILK in promoting pancreatic cancer progression.

## Materials and methods

### Cell line, culture and reagents

Non-malignant human primary pancreatic cells (NPC) were purchased from Applied Biological Materials (Richmond, BC, Canada) and cultured in Prigrow I medium. AsPC-1, BxPC-3, Panc-1, MiaPaCa2 and SW1990 pancreatic cancer cells, DU-145 prostate cancer cells, and MDA-MB-231 breast cancer cells were purchased from American Type Culture Collection (Manassas, VA, USA). AsPC-1, BxPC-3, Panc-1, SW1990 and DU-145 cells were cultured in RPMI 1640 medium, and MiaPaCa2 and MDA-MB-231 cells were cultured in DMEM medium, supplemented with penicillin–streptomycin and 10% fetal bovine serum (Invitrogen, Carlsbad, CA, USA). Cells were cultured at 37 °C in a humidified incubator containing 5% CO_2_. In hypoxia incubation, cells were cultured in hypoxia chamber (Proox 110, BioSpherix, Parish, NY, USA), flushed with 1% O_2_, 5% CO_2_ and 94% N_2_ at 37°C for the indicated time. Cells were recently authenticated by STR profiling and tested to confirm mycoplasma free. The ILK inhibitor T315 was synthesized in our laboratory as described previously.^35^ MG-132, SP-600125, SB-203580 and SB-216763 were purchased from Sigma-Aldrich (St Louis, MO, USA), and Bay-11-7082, cycloheximide and GF-109203X were from Calbiochem (San Diego, CA, USA). Antibodies for various proteins were from the following sources: ILK, Flag, STAT3, p-Tyr-705 STAT3, CREB, α-tubulin, Cell Signaling Technology (Danvers, MA, USA); MUC1-C, β-TrCP, Thermo Fisher Scientific (Fremont, CA, USA); β-actin, MP Biomedicals (Irvine, CA, USA); p-Ser/Thr, Fbw7, Abcam, (Cambridge, MA, USA); ubiquitin, PKCα, p-Ser-657 PKCα, PKCβII, PKCδ, p-Thr-507-PKCδ, PKCε, PKCγ, PPARγ, Santa Cruz Biologicals (Santa Cruz, CA, USA); rabbit anti-mouse IgG-HRP conjugates, goat anti-rabbit IgG-HRP conjugates, goat anti-Armenian hamster HRP conjugates, Jackson ImmunoResearch Laboratories (West Grove, PA, USA). Specific catalog numbers for each antibody are listed in Supplementary Table S1.

### AsPC-1^IL-6^ stable clone selection

AsPC-1 cells were seeded in a 6-cm dish and transiently transfected with the pCMV-IL6 plasmid. After 2 days, cells were expanded into ten 10-cm dishes and incubated with fresh culture medium containing G418 until colonies grew. Each single colony was then trypsinized using a cloning cylinder and expanded. IL-6 secretion into culture medium by cell colonies was analyzed by the IL-6 DuoSet ELISA kit (R&D, Minneapolis, MN, USA) according to the manufacturer’s instructions. One clone that produced the optimal amount of IL-6 in culture medium (550 ng/ml versus nil for parental AsPC-1 cells), designated as AsPC-1^IL-6^, was used in this study.

### Transient transfection and immunoblotting

Cells were transfected with plasmids or siRNAs using Lipofectamine 2000 (Thermo Fisher Scientific) according to the manufacturer’s instructions. pCMV-IL6, Flag-tagged ILK-WT and GFP-tagged ILK (ILK-CA and ILK-KD) plasmids were generated as previously described.^32, 34^ Other plasmids, shRNA and siRNA used in this study and their sources were: pcDNA3.1, Addgene (Cambridge, MA, USA); ILK siRNA (#6202), Cell Signaling Technology (Beverly, MA, USA); E2F1 shRNA, PPARγ siRNA, Santa Cruz Biologicals; Fbw7 siRNA and β-TrCP siRNA, OriGene Technologies Inc. (Rockville, MD, USA). Immunoblotting and densitometric analysis were performed as previously described.^34^

### Lentivirus preparation and transfection

Lentivirial plasmids were cotransfected with the third Generation Packaging Systems (pMDLg/pRRE, #12251; pRSV-Rev, #12253; pMD2.G #12259) (Addgene) in 293T cells following a standard calcium phosphate transfection procedure. Viral particles were collected for transfection of target cells, followed by puromycin selection for 1 week to generate stable cell lines. The following lentiviral plasmids were obtained from Academia Sinica (Taipei, Taiwan): pLAS.Void (negative control), shILK (#0968), shSTAT3 (886, 887), shPKCα (#3511, #6909), shPKCβ1 (#3117, #5381), shPKCδ (#1180, #2640), shPKCε (#0844, #0848) and shPKCγ (#0693, #2326).

### Cellular fractionation

The following buffers were used for nuclear fractionation: harvest buffer (10 mM, 50 mM NaCl, 0.5 M sucrose, 0.1 mM EDTA, 0.5% Triton X-100); buffer A (10 mM HEPES pH 7.9, 10 mM KCl, 0.1 mM EDTA, 0.1 mM EGTA); buffer C (10 mM HEPES pH 7.9, 500 nM NaCl, 0.1 mM EDTA, 0.1 mM EGTA, 0.1% NP-40). Immediately before use, the following was added to each buffer: 2 mM sodium orthovanadate, 5 mM NaF, 1 mM sodium pyrophosphate, 2.5 mM glycerol phosphate, 1 mM DTT, 1 mM PMSF and protease inhibitor. After the indicated treatment, cells were collected and resuspended in cold harvest buffer with 5 min incubation on ice. Cells were centrifuged at 3000 rpm for 10 min and supernatant were collected and centrifuged at 14 000 rpm for 15 min. The supernatants were collected as cytoplasmic proteins and membrane proteins. The pellet was washed using buffer A and resuspended in buffer C. The resuspended pellet was vortexed at high speed for 30 s and incubated on ice for 10 min three times. After centrifugation at 13 000 rpm for 15 min, the supernatant was collected as nuclear proteins.

### qRT-PCR

Total RNA was isolated using TRIzol reagent (Invitrogen) and reverse-transcribed to cDNA using the iScript cDNA synthesis kit (Bio-Rad; Hercules, CA, USA). For qRT-PCR, cDNA was amplified in iQ SYBR Green Supermix (Bio-Rad) and detected with the Bio-Rad CFX96 RT-PCR detection system. Relative gene expression was normalized to GAPDH and calculated by using the 2(^−ΔΔCT^) method. The primer sequences were: MUC1, forward: 5′-ACCTACCATCCTATGAGCGAG-3′, reverse: 5′-GGTTTGTGTAAGAGAGGCTGC-3′ GAPDH: forward: 5′-AGGGGTCTACATGGCAACTG-3′, reverse: 5′-CGACCACTTTGTCAAGCTCA-3′.

### Co-IP analysis

Cells were lysed in lysis buffer (20 mM Tris-HCl pH 7.5, 150 mM NaCl, 1% Triton X-100) containing phosphatase and protease inhibitor mixture on ice for 30 min. After centrifugation at 13 000 *g* for 10 min, one-tenth the volume of the supernatant was stored at 4 °C for use as input, and the remainder was incubated with 20 μl protein A/G-sepharose beads at 4 °C for 1 h to eliminate nonspecific binding. Protein A/G beads were then removed by centrifugation and the supernatants were incubated with primary antibodies at 4 °C overnight with rocking followed by incubating with 20 μl protein A/G-sepharose beads at 4 °C for 2 h. The immunocomplexes were washed with lysis buffer, resuspended in 2 × sample buffer, and then subjected to immunostaining.

### Statistical analysis

Each experiment was performed at least three times. Student’s *t*-test was performed using GraphPad Prism 5 (GraphPad Software, Inc., San Diego, CA, USA) to compare the mean of each group with that of the control group in experiments. *P*-values less than 0.05 were considered significant (*P<0.05, **P<0.01, ***P<0.001).
