# Correction: Role of integrin-linked kinase in regulating the protein stability of the MUC1-C oncoprotein in pancreatic cancer cells

**DOI:** 10.1038/s41389-020-0213-4

**Published:** 2020-02-26

**Authors:** H.-L. Huang, H.-Y. Wu, P.-C. Chu, I.-L. Lai, P.-H. Huang, S. K. Kulp, S.-L. Pan, C.-M. Teng, C.-S. Chen

**Affiliations:** 1 The PhD Program for Cancer Biology and Drug Discovery, College of Medical Science and Technology, Taipei Medical University, Taipei, Taiwan; 2 Division of Medicinal Chemistry and Pharmacognosy, College of Pharmacy, The Ohio State University, Columbus, OH USA; 3 Institute of Biological Chemistry, Academia Sinica, Taipei, Taiwan; 4 Institute of Biochemical Science, National Taiwan University, Taipei, Taiwan; 5 Epigenome Research Center, China Medical University Hospital, Taichung, Taiwan; 6 Department of Biochemistry and Molecular Biology, College of Medicine, National Cheng Kung University, Tainan, Taiwan; 7 Department of Pharmacology, College of Medicine, Taipei Medical University, Taipei, Taiwan; 8 Pharmacological Institute, College of Medicine, National Taiwan University, Taipei, Taiwan

**Correction to: Oncogenesis**

10.1038/oncsis.2017.61 published online 10 July 2017

After publication of the Article, the authors noticed that corrections to the central panel of Fig. 1b (the time-dependent effect of exogenous IL-6 on the expression/phosphorylation of Stat3, ILK, and MUC1-C) and the left panel of Fig. 2a (effect of shRNA-mediated silencing of ILK versus control on the phosphorylation and/or expression of STAT3 and MUC1-C in IL-6-treated AsPC-1 cells) were necessary, as anomalies were found in the Stat3 and ILK blots, respectively. Experiments were repeated outside of the authors’ laboratory. The new data are essentially the same as those acquired previously and lead to the same conclusion reported in the original Article. The corrected figures containing these new data are shown below.

**Figure 1:**
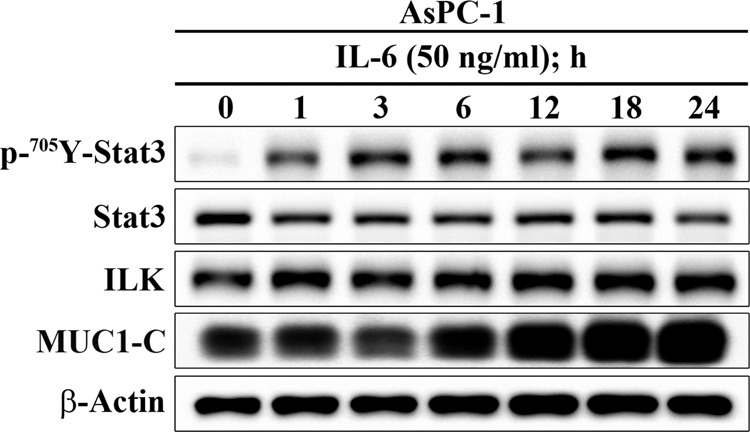
Fig. 1

**Figure 2:**
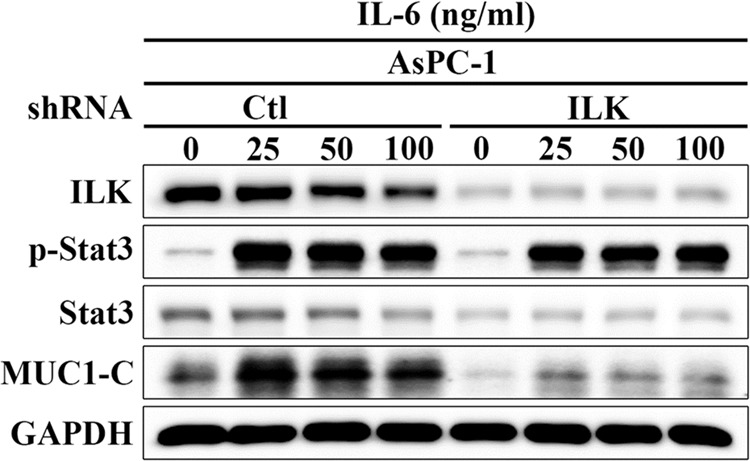
Fig. 2

