# Supplementary material for: Role of integrin-linked kinase in regulating the protein stability of the MUC1-C oncoprotein in pancreatic cancer cells
Source: Oncogenesis. 2017 Jul 10;6(7):e359–. doi: 10.1038/oncsis.2017.61 (PMC5541713; doi:10.1038/oncsis.2017.61)
Supplement: Supplementary Information [file oncsis201761x1.docx]

**Role of Integrin-Linked Kinase in Regulating the Protein Stability of the MUC1-C Oncoprotein in Pancreatic Cancer Cells**

Han-Li Huang^1,2^, Hsing-Yu Wu^3,4^, Po-Chen Chu^2,3^, I-Lu Lai^2,5^, Po-Hsien Huang^6^, Samuel K. Kulp^2^, Shiow-Lin Pan^1,7^, Che-Ming Teng^8^, Ching-Shih Chen^2,3^

**Supplementary Information**

**Supplementary Fig. S1**. Suppressive effect of siRNA-mediated knockdown of ILK on MUC1-C expression in DU-145 and MDA-MB-231 cells.

**Supplementary Fig. S2**. Suppressive effect of T315 (2 µM) on hypoxia-induced MUC1-C upregulation in AsPC-1 cells under hypoxic conditions for 24 and 48 h.
