# Supplementary material for: Role of integrin-linked kinase in regulating the protein stability of the MUC1-C oncoprotein in pancreatic cancer cells
Source: Oncogenesis. 2017 Jul 10;6(7):e359–. doi: 10.1038/oncsis.2017.61 (PMC5541713; doi:10.1038/oncsis.2017.61)
Supplement: Supplementary Table S1 [file oncsis201761x2.docx]

**Supplementary Table S1. Information of antibodies used in Western blotting**

| **Antibodies Vendor Catalog number** | | |
| --- | --- | --- |
| Anti-ILK | Cell Signaling Technology, Danvers, MA, USA | 3862 |
| Anti-Flag | Cell Signaling Technology, Danvers, MA, USA | 14793 |
| Anti-Tyr-705 STAT3 | Cell Signaling Technology, Danvers, MA, USA | 9145 |
| Anti-STAT3 | Cell Signaling Technology, Danvers, MA, USA | 9139 |
| Anti-CREB | Cell Signaling Technology, Danvers, MA, USA | 9197 |
| Anti-α-tubulin | Cell Signaling Technology, Danvers, MA, USA | 2144 |
| Anti-MUC1-C | Thermo Fisher Scientific, Fremont, CA, USA | HM-1630-P1 |
| Anti-β-TrCP | Thermo Fisher Scientific, Fremont, CA, USA | 37-3400 |
| Anti-β-actin | MP Biomedicals, Irvine, CA, USA | 08A000060 |
| Anti-p-Ser/Thr | Abcam, Cambridge, MA, USA | Ab17464 |
| Anti-Fbw7 | Abcam, Cambridge, MA, USA | Ab171961 |
| Anti-ubiquitin | Santa Cruz Biotechnology, Santa Cruz, CA, USA | sc-8017 |
| Anti-PKCα | Santa Cruz Biotechnology, Santa Cruz, CA, USA | sc-8393 |
| Anti-Ser-657 PKCα | Santa Cruz Biotechnology, Santa Cruz, CA, USA | sc-377565 |
| Anti-PKCβII | Santa Cruz Biotechnology, Santa Cruz, CA, USA | sc-210 |
| Anti-PKCδ | Santa Cruz Biotechnology, Santa Cruz, CA, USA | sc-937 |
| Anti-p-Thr-507 PKCδ | Santa Cruz Biotechnology, Santa Cruz, CA, USA | sc-365969 |
| Anti-PKCε | Santa Cruz Biotechnology, Santa Cruz, CA, USA | sc-214 |
| Anti-PKCγ | Santa Cruz Biotechnology, Santa Cruz, CA, USA | sc-211 |
| Anti-PPARγ | Santa Cruz Biotechnology, Santa Cruz, CA, USA | sc-7273 |
| Rabbit anti-mouse IgG-HRP conjugates | Jackson ImmunoResearch Laboratories, West Grove, PA, USA | 115-035-003 |
| Goat anti-rabbit IgG-HRP conjugates | Jackson ImmunoResearch Laboratories, West Grove, PA, USA | 111-035-003 |
| Goat anti-Armenian hamster HRP conjugates | Jackson ImmunoResearch Laboratories, West Grove, PA, USA | 127-035-160 |
